# Proposed use of spatial mortality assessments as part of the pesticide evaluation scheme for vector control

**DOI:** 10.1186/1475-2875-12-366

**Published:** 2013-10-18

**Authors:** Beatriz Mosqueira, Joseph Chabi, Fabrice Chandre, Martin Akogbeto, Jean-Marc Hougard, Pierre Carnevale, Santiago Mas-Coma

**Affiliations:** 1 Departamento de Parasitologia, Facultad de Farmacia, Universidad de Valencia, Av Vicent Andrés Estellés s/n, Burjassot 46100, Valencia, Spain; 2 Centre de Recherches Entomologique de Cotonou, 06 BP 2604, Cotonou, Benin; 3 Institut de Recherche pour le Développement (IRD), Laboratoire de Lutte contre les Insectes Nuisibles, BP 64501, Montpellier Cedex 5 34394, France; 4 Immeuble Le Majoral, Avenue de la Tramontane, Portiragnes Plage 34420, France

**Keywords:** Vector control, WHOPES, Insecticide-treated nets (ITNs), Long-lasting insecticidal nets (LLINs), Indoor residual spraying (IRS), Insecticide paint, Mass effect

## Abstract

**Background:**

The WHO Pesticide Evaluation Scheme to evaluate the efficacy of insecticides does not include the testing of a lethal effect at a distance. A tool was developed to evaluate the spatial mortality of an insecticide product against adult mosquitoes at a distance under laboratory and field conditions. Operational implications are discussed.

**Methods:**

Insecticide paint, Inesfly 5A IGR™, containing two organophosphates (OPs): chlorpyrifos and diazinon, and one insect growth regulator (IGR): pyriproxyfen, was the product tested. Laboratory tests were performed using “distance boxes” with surfaces treated with one layer of control or insecticide paint at a dose of 1 kg/6 sq m. Field tests were conducted up to 12 months in six experimental huts randomly allocated to control or one or two layers of insecticide paint at 1 kg/6 sq m. All distance tests were performed using reference-susceptible strains of *Anopheles gambiae* and *Culex quinquefasciatus* left overnight at a distance of 1 m from control or treated surfaces.

**Results:**

After an overnight exposition at distances of 1 m, field and laboratory evaluations at 0 months after treatment (T0) yielded 100% mortality rates on surfaces treated with one layer at 1 kg/6 sq m against susceptible strains of *An. gambiae* and *Cx. quinquefasciatus*. Testing for long-term efficacy in the field gave mortality rates of 96-100% after an overnight exposition at a distance of 1 m for up to 12 months in huts where a larger volume was treated (walls and ceilings) with one or two layers of insecticide paint.

**Conclusion:**

A comprehensive evaluation of the full profile of insecticide products, both upon contact and spatially, may help rationalize vector control efforts more efficiently. Treating a large enough volume may extend a product’s mortality efficacy in the long-term, which contact tests would fail to assess. It is hereby proposed to explore the development of cost effective methods to assess spatial mortality and to include them as one additional measurement of insecticide efficacy against mosquitoes and other arthropod vectors in WHOPES Phase I and Phase II studies.

## Background

Vector-borne diseases, such as malaria and dengue, are among the major causes of morbidity and mortality and significantly impede the economic and social development of many countries, predominantly in tropical areas, although not only. In temperate regions, West Nile virus, dengue, leishmaniasis and chikungunya, among other vector-borne diseases, are also causing an increasing burden.

Control strategies rely mostly on vector control using insecticides, treatment using drugs, improving people’s dwellings/modifying the environment, education, and the creation of new vaccines. A promising intervention strategy involves genetic control of the vectors [1]. The strategies chosen will depend on several factors, such as resistance to insecticides, availability of treatment and/or resistance to available drugs, difficulties in developing a vaccine, existence of operational genetic control programmes, and long-term sustainability. A combination of the above disease control strategies will increase chances to succeed.

Vector control is one of these strategies and remains a key player in the control of major endemic and epidemic vector-borne diseases such as malaria [2,3]. The official World Health Organization Pesticide Evaluation Scheme (WHOPES) guidelines for the evaluation of the efficacy of insecticides [4,5] take into consideration products’ impact on mortality, blood feeding, deterrence and repellence. Tests currently used include classical WHO contact bioassays [6,7], tunnel tests [8,9] and early morning collections in experimental huts [10,11]. These tests provide key information on the impact of insecticide products, such as long-lasting insecticidal nets (LLINs) or indoor residual spraying (IRS), upon contact both in the laboratory and the field, but does not provide information on the possible lethal effect at a distance.

Since even highly endophilic mosquitoes or other arthropod vectors are not always in contact with an insecticide-treated surface before biting a human or animal host, especially on pyrethroid-treated surfaces due to its irritant effect, it is desirable to evaluate the lethal effect spatially, that is, at a distance, without the mosquitoes ever entering into contact with an insecticide-treated surface.

Several studies on the community effect of ITNs on malaria indicate the presence of a beneficial mass effect [12-20]. A mass effect of IRS has also been documented in a number of trials [21].

In this study, distance tests were performed in the laboratory using “distance boxes”, and in the field. In the field, evaluations were done in addition to WHO bioassays and early morning collections in experimental huts. The product evaluated consisted of an insecticide paint, Inesfly 5A IGR™, composed of two organophosphates (OPs): chlorpyriphos (1.5%) and diazinon (1.5%), and an insect growth regulator (IGR): pyriproxyfen (0.063%). The product was a white vinyl paint with an aqueous base. Active ingredients resided within Ca CO^3^ + resin microcapsules ranging from one to several hundred micrometres in size. The formulation allows a gradual release of active ingredients, increasing its durability. Toxicology studies performed so far support the product’s safety [22-24]. Inesfly 5A IGR™ had been evaluated previously under experimental conditions against the Chagas disease vector *Triatoma infestans*[25,26], Classical WHOPES tests were also performed on Inesfly 5A IGR™ in the laboratory (Phase I) against 100% OP-resistant *Culex quinquefasciatus*[27] and in the field (Phase II) against local wild pyrethroid-resistant populations of the major malaria vector, *Anopheles gambiae*, and pest mosquito, *Cx. quinquefasciatus*[28]. In parallel to the standard Phase I evaluations [27], it was decided to explore the idea of a possible efficacy at a distance by exposing mosquitoes to metal-treated surfaces at distances of 3 cm, 40 cm and 100 cm. Mortalities at shorter distance were almost the same as the ones upon contact (unpublished results). It was thus decided to test spatial mortality at distances of 100 cm from cement-treated surfaces so as to reproduce the same test on experimental huts during Phase II evaluations. The objective of this paper is to propose the use of spatial mortality tests as part of the WHOPES in the light of results obtained in the laboratory (Phase I) using “distance boxes” and in the field (Phase II) in experimental huts.

## Methods

### Phase I - laboratory tests using distance boxes

Two identical wooden boxes were built, one for control and one for treatment. The size of each wooden box was 50 cm wide × 50 cm high, length 100 cm with two horizontal slits of 4 cm × 50 cm on each side. The two horizontal slits were placed in the middle of each side of the box to allow air to flow. Wood was chosen as a material readily available and easy to work with. One end was left open and is where mosquitoes were placed inside 150 ml tubes. The other end was closed by a cement surface 50 cm × 50 cm – cement was chosen to reproduce the material experimental huts were made of. The box used as control had a cement surface with no paint. The box used for treatment had a cement surface with of one layer of Inesfly 5A IGR™ insecticide-paint at 1 kg/6 sq m. Boxes were placed in a closed room at 80 ± 10% relative humidity and 27 ± 2°C temperature.

Unfed females of *An. gambiae* Kisumu and *Cx. quinquefasciatus* S-Lab, three to five days old, reared at the Centre de Recherche Entomologique de Cotonou (CREC) insectarium, and susceptible to all insecticides, were used. Mosquitoes were introduced in four 150 ml tubes with mosquito netting at both ends to protect them from scavengers but allow air through. Honey juice-soaked cotton was introduced in each tube to prevent females from starvation. Four replicates were made with 15 females each, giving a total of 60 females per surface per test. Tubes were placed horizontally at the edge of the box at 1 m from the cement surface from 19:00 to 07:00. The following morning, females were taken to the insectarium for delayed mortality assessments after 24 hours at 80 ± 10% relative humidity and 27 ± 2°C temperature. Distance testing was done only at 0 months after treatment (T0) under laboratory conditions.

### Phase II - field tests in experimental huts in Benin

Inesfly 5A IGR™ was evaluated in six experimental huts at the Ladji station in Cotonou (south of Benin) [28]. Experimental huts were built following the West African-style hut model [29]. Huts were treated with one or two layers of insecticide paint at 1 kg commercial product/6 sq m, that is 0,51 g a.i. per sq m. Based on huts’ dimensions, 3.4 kg of paint were applied on walls per layer, and 1.0 kg on ceilings. Huts treated with two layers had the first layer diluted in 20% water following recommendations of the manufacturer. The overall random disposition of huts was: H1: Control 1 - no paint; H2: Control 2 - two layers of control paint on walls and ceiling; H3: one layer of insecticide paint on walls; H4: one layer of insecticide paint on walls and ceiling; H5: two layers of insecticide paint on walls; and H6: two layers of insecticide paint on walls and ceiling.

Unfed females of *An. gambiae* Kisumu and *Cx. quinquefasciatus* S-Lab, three to five days old, reared at the CREC insectarium, and susceptible to all insecticides, were used. A total of 60 females were introduced into four tubes of 150 ml, with 15 females per tube. Mosquito netting was placed at both ends to allow air through. Honey-soaked cotton was introduced to ensure that females did not die from starvation. Tubes containing females were placed inside the hut, on the floor, horizontally from 19:00 to 07:00, at a distance of 1 m from two perpendicular walls inside the hut and 1.90 m from the ceiling. The following morning, females were taken to the insectarium for delayed mortality assessment after 24 hours at 80 ± 10% relative humidity and 27 ± 2°C temperature. Tests were performed again 12 months after treatment.

Results from laboratory and field distance tests were analysed using Epi-Info 6. When values were <5, Fisher exact tests were used.

## Results

### Phase I - laboratory tests using distance boxes

Distance boxes yielded 100% mortality at 0 months after treatment against both *An. gambiae* Kisumu and *Cx. quinquefasciatus* S-Lab (Table 1). Compared to control, mortality rates were significantly different for the treated surface (p < 10^-6^).

**Table 1:** Comparison of phase I and phase II spatial mortality rates in control and treated surfaces

**Cement tested at a distance of 1 m at T0**	**Control**	**One layer IP at 1 kg/6 sq m in distance box – phase I**	**One layer IP at 1 kg/6 sq m in experimental huts - phase II**
*An. gambiae* Kisumu	0^a^	100^b^	100^b^
*Cx. quinquefasciatus* S-Lab	0^a^	100^b^	100^b^

Delayed 24-hour mortality of *Anopheles gambiae* Kisumu and *Culex quinquefasciatus* S-Lab after overnight exposure at a distance of 1 m in distance boxes in laboratory, and in experimental huts in the field.

*IP,* Insecticide paint, *T0*, 0 months after treatment. Values in the same row sharing a letter superscript do not differ significantly (P ≥ 0.05).

### Phase II - field tests in experimental huts in Benin

Under field conditions at T0, all huts, regardless of the surface treated and the number of layers, yielded 100% mortality against both *An. gambiae* Kisumu and *Cx. quinquefasciatus* S-Lab (Table 1). Twelve months after treatment, mortality rates observed at the huts where a larger volume was treated with one or two layers of paint were 98.4% for *An. gambiae* Kisumu and 96.2% for *Cx. quinquefasciatus* S-Lab (Table 2). Mortality rates in the hut treated on only walls with one layer of insecticide paint was lower than in the other three huts: 36% mortality against susceptible *An. gambiae* Kisumu and 60% against susceptible *Cx. quinquefasciatus* S-Lab (p < 10^-6^), though still higher than control (p < 10^-6^).

**Table 2:** Phase II spatial long-term mortality rates in control and treated experimental huts

**Phase II - cement tested at a distance of 1 m at T0 and T12**	**Timepoint**	**Control 1**	**Control 2 two layers of control paint on walls and ceiling**	**One layer IP on walls at 1 kg/6 sq m**	**One layer IP on walls and ceiling at 1 kg/6 sq m**	**Two layers IP on walls at 1 kg/6 sq m**	**Two layers IP on walls and ceiling at 1 kg/6 sq m**
*An. gambiae*	T0	0^a^	3.4^a^	100^b^	100^b^	100^b^	100^b^
Kisumu	T12	1.5^a^	3^a^	35.6^b^	98.4^c^	100^c^	100^c^
*Cx. quinquefasciatus* S-Lab	T0	8.3^a^	0^a^	100^b^	100^b^	100^b^	100^b^
	T12	1.8^a^	3^a^	60^b^	96.2^c^	100^c^	100^c^

Delayed 24-hour mortality of *Anopheles gambiae* Kisumu and *Culex quinquefasciatus* S-Lab after overnight exposure at a distance of 1 m from two perpendicular walls in experimental huts in the field.

*IP*, Insecticide paint, *T0* and *T12*, 0 and 12 months after treatment.

Values in the same row sharing a letter superscript do not differ significantly (P ≥ 0.05).

## Discussion

The results obtained in the laboratory and the field were similar at T0: 100% mortality rates were observed on surfaces treated with one layer of insecticide paint at the recommended dose of 1 kg/6 sq m against susceptible *An. gambiae* Kisumu and *Cx. quinquefasciatus* S-Lab. Female mosquitoes were never in contact with the treated or control surfaces. A 1-m distance was respected in all cases for all repeats. The uniformity of results suggests distance boxes could be a useful and simple approach for testing the lethal efficacy of insecticide products at a distance in the laboratory during Phase I evaluations, but more data is needed. The distance of one metre was chosen because of the small size of experimental huts and of West African homes in general. The distance of one metre is thus proposed as an initial step, nevertheless the distance may be adapted depending on the nature of the insecticide (ie. vapour pressure) and the support used (ie. LLINs, IRS, DL, paint). The size of the dwellings to be treated may also play a role in deciding the distance to be tested – if large halls in schools, airports or hospitals are treated, it may be of interest to test for spatial mortality efficacy at greater distances.

To test for long-term spatial mortality efficacy, distance tests were performed again 12 months after treatment during Phase II studies in the field: spatial mortality after an overnight exposition at distances of 1 m remained high, 96-98%, against susceptible *An. gambiae* Kisumu and *Cx. quinquefasciatus* S-Lab in huts with one layer of insecticide paint if both, walls and ceiling were treated. On the other hand, huts treated with one layer but on walls only (not ceilings) performed less well, 36% mortality against susceptible *An. gambiae* Kisumu and 60% against susceptible *Cx. quinquefasciatus* S-Lab after 12 months. That is, provided that a large enough volume was treated, huts with one layer performed as well as huts with two layers despite the difference in dose – this finding was referred to as the “volume effect”. This notion of volume effect seemed to be supported by results obtained during Phase II early morning collections in the field: A volume effect was observed during early morning collections performed in experimental huts in Ladji, south of Benin, with the same insecticide paint [28]. Likewise, a volume effect was observed when testing the efficacy of a pyrethroid-based insecticide paint against pyrethroid-susceptible populations of *An. gambiae* and *Cx. quinquefasciatus* during a Phase II study in experimental huts in the north of Benin: female mosquitoes exposed at a distance of 1 m from treated surfaces had a significantly higher mortality rate 12 months after treatment in huts where both walls and ceiling were treated even if with just one layer of paint, but not in huts with one layer on walls only [Mosqueira B, Chabi J, Soukou KB, Akogbeto M, Carnevale P, Corbel V, Mas-Coma S: Laboratory and field efficacy of a pyrethroid-based insecticide paint against insecticide-susceptible and resistant malaria-transmitting mosquitoes, in preparation]. Curiously, an irritant and deterrent effect was observed when comparing treated huts to control. Predictably, WHO contact bioassays failed to detect a volume effect: as far as WHO contact bioassays went, only the dose applied counted – two layers performing consistently better than one layer. Hence, Phase I and Phase II efficacy assessments based on contact may be overlooking important questions on the coverage and dose needed to achieve long-term efficacy such as: would reducing the total dose be possible if a larger surface was treated? Phase II early morning collections do offer an insight on the question, as not all wild mosquitoes entering the hut are in direct contact with treated surfaces, but the exact distances from treated surfaces would not be known, numbers would vary between huts and whether the insecticide is on the walls or bed nets might make a difference. Likewise, current Phase I and Phase II assessments may not fully explore the potential of high vapour pressure insecticides by evaluating efficacy chiefly upon contact as opposed to both, contact and distance.

The tested insecticide product was effective in killing mosquitoes that had not come closer than 1 m to the treated surface after an overnight exposition. It could be envisaged that the same could happen in a natural setting to mosquitoes resting on non-treated surfaces before and/or after biting, although it is not known the minimum amount of exposure time needed to achieve this. A study performed by Gimnig *et al.*[17] determined how the abundance of malaria vectors changed as a function of distance from houses with ITNs. The study used a geographic information system (GIS) to test the hypothesis that a community effect reduces the overall vector population, and that persons lacking ITNs who live near the compounds of those using ITNs are afforded some protection from vector mosquitoes. Another study performed by Hawley *et al.*[30] showed ITNs had a protective effect on child mortality, moderate anaemia, high-density parasitaemia, and haemoglobin levels in compounds lacking ITNs but located within 300 m of compounds with ITNs. A mass community effect against malaria transmission has also been observed in areas where the only malaria vector is largely exophagic and zoophilic [31].

In the case of dengue, a spatial analysis performed by Lenhart *et al.*[32] indicated that the effect of the presence of ITNs had spread to control houses located 50–100 m away from bed net houses by five months post-intervention, although control houses located more than 100 m from bed net houses experienced no significant change in entomological indices. Findings were particularly surprising since there were no previous indications that ITNs could be useful in reducing dengue transmission. Although the nature of that effect was not characterized, the study suggested that it was of both repellent and lethal nature and not by the barrier provided by the ITN itself [32].

Findings increasingly suggest the mass community effect depends on high rates of coverage [33,34] as well as the distance from treated clusters [35]. Despite evidence pointing at the mass community effect of vector control strategies, little is known on the exact mechanism. Contact alone may not be the sole factor. Recent findings emphasize the need to study the spatial repellency of insecticides in addition to the contact irritancy tests classically done [36]. Furthermore, spatial repellency may represent an effective tool in the fight against vector-borne disease transmission [37]. It is proposed that the spatial *mortality* of insecticide products is evaluated in addition to the contact mortality tests currently done in order to better rationalize vector control efforts. Future endeavours will be directed towards the testing of the lethal efficacy at a distance of Inesfly 5A IGR™ against OP-resistant *An. gambiae* in the laboratory and the field.

## Conclusions

Spatial mortality assessments provide additional information overlooked by the contact efficacy tests recommended at present. It is therefore proposed that tests to evaluate the spatial mortality effect of insecticides are added to the battery of Phase I and Phase II WHOPES tests in the laboratory and the field. A tool to evaluate an insecticide product’s lethal effect at a distance in the laboratory may be distance boxes although further studies are needed before this method is standardised. In the field, exposing mosquitoes at a fixed distance from treated surfaces may provide valuable information with little added effort. In order to better rationalize integrated vector control strategies, it may be important to assess the full profile of an insecticide by doing both contact and spatial lethal efficacy tests.

## Competing interests

The authors declare that they have no competing interests.

## Authors’ contributions

SMC had the idea of performing distance tests as part of the protocol to test for insecticide efficacy. SMC, PC and BM conceived the protocol. PC, SMC and BM contributed to the design of the study. FC and JMH contributed to the implementation of the study. JC and BM conducted evaluations. MA was the director of the Centre de Recherche Entomologique de Cotonou (CREC). The manuscript has been written by BM and has been revised by SMC and JC. All authors read and approved the final manuscript.
